# Oyster-derived Zinc Exhibits Superior Anti-anemic Efficacy and Bioavailability Compared with Conventional Zinc Supplements

**DOI:** 10.1007/s12011-026-05073-x

**Published:** 2026-03-25

**Authors:** Yen-Hua Chen, Hui-Lin Feng, Jia-Ling Tang, Chen-Wei Chang, Sen-Shyong Jeng

**Affiliations:** 1 Institute of Food Safety and Risk Management, College of Life Sciences, National Taiwan Ocean University, Keelung, 202301 Taiwan; 2 Master Program of Food Safety Management, College of Life Sciences, National Taiwan Ocean University, Keelung, 202301 Taiwan; 3 Department of Food Science, College of Life Sciences, National Taiwan Ocean University, Keelung, 202301 Taiwan

**Keywords:** Anemia, Bioavailability, Caco-2 cells, Oyster, Zinc, Zinc deficiency

## Abstract

Zinc is an essential trace element involved in numerous biological processes. Zinc bioavailability is a central issue in nutritional biochemistry; however, comparative evidence linking zinc source, intestinal absorption, and physiological efficacy remains limited. In this study, we compared the bioavailability and anti-anemic efficacy of zinc derived from oysters (*Crassostrea gigas*) with those of conventional zinc supplements. Using a phenylhydrazine-induced hemolytic anemia rat model, we demonstrate that oral supplementation with oyster-derived zinc effectively restores red blood cell counts. Selective chelation of zinc from oyster tissue using 1,10-phenanthroline abolished this effect, confirming zinc as the principal active component. Dose–response analysis identified a minimum effective dose of 2.1 mg Zn/rat/day for oyster-derived zinc, whereas a substantially higher dose of zinc sulfate (5.0 mg Zn/rat/day) was required to achieve comparable erythropoietic recovery. When equivalent zinc doses were administered, only oyster-derived zinc significantly increased plasma erythropoietin (EPO) levels and restored erythropoiesis. In vitro studies using simulated gastrointestinal digestion and Caco-2 cell transport assays revealed that oyster-derived zinc exhibits approximately two-fold higher bioavailability than inorganic zinc (zinc sulfate), organic zinc salts (zinc gluconate or zinc acetate), or peptide-bound zinc (polaprezinc). Collectively, these findings demonstrate that zinc delivered within a natural food matrix confers enhanced bioavailability and biological efficacy, highlighting oyster-derived zinc as a nutritionally advantageous source for alleviating zinc deficiency and correcting zinc-responsive anemia.

**Supplementary Information:**

The online version contains supplementary material available at 10.1007/s12011-026-05073-x.

## Introduction

Zinc is an essential trace element required for numerous biological processes in humans [1]. In addition to its well-established structural and catalytic roles in a wide range of proteins, increasing evidence indicates that zinc ions also function as signaling molecules that regulate cellular processes and gene expression [2]. One emerging biological role of zinc signaling is the stimulation of erythropoiesis. Supplementation with additional zinc—administered either orally or by injection—has been shown to promote red blood cell production in several animal models, including fish and rodents [3–6]. This phenomenon, referred to as zinc-induced erythropoiesis, highlights zinc as a nutritionally relevant modulator of hematopoietic function [7].

Anemia remains the most common blood disorder worldwide and arises from diverse causes, including nutritional deficiencies (iron, vitamin B₁₂, folate), chronic kidney disease (CKD), and bone marrow dysfunction [8–11]. Current therapeutic strategies focus primarily on correcting nutrient deficiencies and, in more severe cases, administering erythropoiesis-stimulating agents, such as recombinant human erythropoietin (rHuEPO) [12–14]. However, accumulating evidence suggests that zinc supplementation may provide an additional nutritional approach to support erythropoiesis under anemic conditions.

Consistent with findings from animal studies, several clinical investigations have demonstrated that zinc supplementation can improve hematologic outcomes. Polaprezinc, an orally bioavailable chelate of zinc and L-carnosine approved for the treatment of gastric ulcers, has been reported to increase hemoglobin levels and reduce ESA requirements in patients with end-stage renal disease when administered at 34 mg elemental zinc per day [15, 16]. Other clinical trials have shown that oral zinc sulfate or zinc acetate hydrate (50 mg Zn/day) improves hemoglobin concentrations or reduces ESA use in CKD patients [17–19], whereas zinc gluconate at 30 mg Zn/day failed to produce comparable effects [20]. Together, these animal and clinical observations indicate that both zinc formulation and dosage critically influence capacity to support erythropoiesis under anemic conditions [21].

The biological efficacy of zinc supplementation depends not only on the administered dose but also on the bioavailability of the zinc source, which is influenced by gastrointestinal digestion, release from the food matrix, intestinal uptake, and systemic transport [22–25]. Accordingly, selection of zinc sources with optimal bioavailability is essential for maximizing nutritional and physiological benefits. The combined use of simulated gastrointestinal digestion and the Caco-2 cell absorption model, which mimics human small-intestinal enterocytes, provides a robust and widely accepted in vitro approach for evaluating zinc bioavailability [25, 26].

To date, three major categories of oral zinc supplements have been evaluated for their anti-anemic effects: inorganic zinc salts (e.g., zinc sulfate), organic zinc salts (e.g., zinc gluconate or zinc acetate), and peptide-bound zinc (polaprezinc). In our previous work, oral supplementation with whole oysters (*Crassostrea gigas*) corrected anemia more effectively than zinc sulfate in rat models of phenylhydrazine (PHZ)-induced hemolytic anemia and 5/6 nephrectomy-induced renal anemia [6]. However, because whole oysters were used, it remained unclear whether the observed effects were attributable solely to zinc or whether other oyster components contributed synergistically.

In the present study, oysters were used as a natural zinc source to compare their anti-anemic efficacy with conventional zinc supplements. To directly assess the contribution of zinc, 1,10-phenanthroline was employed to selectively chelate zinc from oyster tissue, generating oyster preparations with graded zinc content. Using these preparations, we identified the minimum effective oral zinc dose required to restore erythropoiesis and compared oyster-derived zinc with commonly used zinc formulations, including zinc sulfate, zinc gluconate, zinc glycinate, and polaprezinc. To determine whether differences in anti-anemic efficacy reflect differences in zinc bioavailability, the relative bioavailability of oyster-derived zinc and other zinc sources was evaluated using simulated gastrointestinal digestion and Caco-2 cell absorption assays. Finally, plasma erythropoietin (EPO) levels were measured to elucidate the mechanistic link between zinc bioavailability and erythropoietic activation.

## Materials and Methods

### Materials

Fresh oysters (*Crassostrea gigas*) were obtained from a certified local supplier, transported on ice, rinsed with deionized water, homogenized, and either used fresh or stored at − 80 °C for freeze-drying. Freeze-dried tissue was milled to a fine powder and stored at − 20 °C. PHZ hydrochloride, 1,10-phenanthroline, and 1,7-phenanthroline were purchased from Sigma–Aldrich (St. Louis, MO, USA). Zinc sulfate (ZnSO₄·7 H₂O), zinc gluconate, zinc glycinate, and polaprezinc (Zn-L-carnosine complex) were of analytical grade.

### Animals

Eight-week-old male Sprague–Dawley rats (210 ± 20 g) were purchased from BioLASCO Taiwan Co., Ltd. Animals were housed under controlled conditions (22 ± 3 °C; 55 ± 5% humidity; 12-h light/dark cycle) with free access to food and water. After one week of acclimatization, body weight averaged 324 ± 13 g. All procedures were approved by the Institutional Animal Care and Use Committee of National Taiwan Ocean University (Approval No. 111019).

### Preparation of Oyster Tissue with Graded Zinc Levels and Oyster-containing Pellets

Fresh oyster homogenates were extracted with 1,10-phenanthroline to selectively deplete zinc. Briefly, 1 g of fresh oyster tissue was extracted with two volumes of 4 mM 1,10-phenanthroline solution (pH 5.0) for 10 min at room temperature, followed by centrifugation at 8,000 × g for 10 min. The zinc content of untreated fresh oyster tissue was determined to be approximately 90 µg Zn/g fresh tissue. Sequential extraction cycles generated oyster tissues containing 30, 43, 50, 70, or 90 µg Zn/g fresh tissue (the latter without extraction). Zinc concentrations were quantified by atomic absorption spectrophotometry following wet digestion with nitric and sulfuric acids. Freeze-dried oyster powder (approximately 3.6 g), corresponding to 30 g fresh oyster tissue, was prepared from oyster tissues containing different zinc levels. The powder was mixed with 3.5% cellulose and 15% water and compressed into compact pellets weighing approximately 4.3 g, hereafter referred to as oyster-containing pellets. Each rat received one oyster-containing pellet daily, providing an additional 0.9, 1.3, 1.5, 2.1, or 2.7 mg Zn/day, depending on the zinc content of the oyster tissue.

### Basal Diet and Zinc Supplement–containing Diet Preparation

The basal diet used in this study was a milk fat globule (MFG) diet, which provides balanced nutrition and has been widely used in dietary intervention studies [27, 28]. Diets were supplied by Oriental Yeast Co., Ltd. (Tokyo, Japan), and the detailed composition of the basal diet is provided in Supplementary Table 1.

Because zinc sulfate, zinc gluconate, and zinc glycinate are highly water-soluble, diets containing these zinc formulations were prepared by applying 0.5 mL of an aqueous zinc solution (1.8 mg Zn/mL) onto a 3 g pellet of the MFG diet. This corresponded to 7.92 mg ZnSO₄·7 H₂O, 12.5 mg zinc gluconate, or 6.38 mg zinc glycinate per milliliter. Each pellet therefore provided 0.9 mg Zn, and each rat received three pellets daily, supplying a total of 2.7 mg Zn/day. Because polaprezinc is poorly soluble in water, 12 mg polaprezinc was mixed with 2 g of powdered MFG diet to provide 2.7 mg Zn per pellet. The mixture was combined with 3.5% cellulose and 15% water and compressed into granulated pellets weighing approximately 2.3 g. Each rat received one such pellet daily.

Under normal conditions, rats consumed approximately 30 g of the basal MFG diet per day. To ensure complete intake of the zinc-supplemented diets, zinc-containing pellets or oyster-containing pellets were administered prior to offering the basal diet. After complete consumption of the supplemented pellets, the basal MFG diet was provided *ad libitum*. No aversion to any zinc-supplemented or oyster-containing diets was observed; pellets were typically consumed within the first 1 h of presentation.

### Induction of Hemolytic Anemia

Hemolytic anemia was induced by a single intraperitoneal injection of PHZ (60 mg/kg body weight) dissolved in saline. Two days after PHZ administration, red blood cell (RBC) counts declined from 7.02 ± 0.58 × 10⁶/mm³ in normal rats (*n* = 38) to 5.01 ± 0.79 × 10⁶/mm³ in PHZ-treated rats (*n* = 38), corresponding to 72 ± 12% of normal values (*p* < 0.001), after which animals were randomized into treatment groups.

### Zinc Supplementation Protocols

PHZ-treated rats were supplemented with zinc from different sources across five independent experimental series, each designed to address a distinct experimental question. All five experimental series involved four days of zinc supplementation prior to sacrifice and analysis.

(i) Dose–response study using oyster tissue (Fig. 2). Rats received oyster-containing pellets providing 0, 0.9, 1.3, 1.5, 2.1, or 2.7 mg Zn/rat/day (n = 12, 6, 6, 12, 12, and 18, respectively).
(ii) Comparison of zinc formulations at a fixed zinc dose (Fig. 3). Rats received either basal diet alone (control) or basal diet containing 2.7 mg Zn/rat/day of ZnSO₄, zinc gluconate, zinc glycinate, polaprezinc, or oyster-containing pellets (n = 40, 18, 29, 23, 29, and 40, respectively).
(iii) EPO–RBC relationship study using graded oyster zinc levels (Fig. 6). Rats were supplemented with oyster-containing pellets providing 0, 0.9, 1.4, 2.1, or 2.7 mg Zn/rat/day (n = 6 per group).
(iv) Comparison of zinc formulations for EPO induction (Fig. 7). Rats received basal diet alone (control) or basal diet containing 2.7 mg Zn/rat/day of ZnSO₄, zinc gluconate, zinc glycinate, polaprezinc, or oyster-containing pellets (n = 18, 6, 6, 6, 6, and 30, respectively).
(v) ZnSO₄ dose–response study (Fig. 8). Rats were supplemented with ZnSO₄-containing basal diet providing 0, 2.7, 3.5, 5.0, or 10 mg Zn/rat/day (n = 12, 6, 6, 12, and 6, respectively).

The five experimental series were conducted sequentially, each addressing a distinct research question (dose–response, formulation comparison, mechanistic correlation, etc.). Sample sizes were adjusted based on preliminary results and statistical power considerations from preceding experiments. In particular, larger group sizes were allocated to the oyster-derived zinc and ZnSO₄ groups because these served as primary comparison arms central to the study hypothesis.

### Simulated Gastrointestinal Digestion

Simulated gastrointestinal digestion was performed to assess zinc digestibility from ZnSO₄, zinc gluconate, zinc glycinate, polaprezinc, and oyster tissue. Each sample contained approximately 2 mg elemental zinc. Gastric and intestinal digestion phases were carried out sequentially under standardized conditions [29]. After digestion, samples were centrifuged to separate digestible and undigested fractions. Zinc content in each fraction was quantified, and digestibility was calculated as the percentage of digestible zinc relative to total zinc.

### Caco-2 Cell Zinc Absorption Assay

Caco-2 cells were cultured and differentiated on Transwell^®^ inserts (24 mm diameter, 0.4 μm pore size; Costar Corp., USA) to form polarized monolayers. Digestible fractions from simulated digestion of ZnSO₄, zinc gluconate, zinc glycinate, polaprezinc, and oyster tissue were adjusted to 25 µg Zn/mL and applied to the apical chamber. Zinc transported to the basolateral chamber was measured, and zinc absorption rate was calculated as the percentage of zinc transported relative to the total zinc applied apically.

### Hematological and Biochemical Analyses

After four days of supplementation, rats were anesthetized and blood samples were collected via cardiac puncture. RBC counts and other hematological parameters were measured using an automated hematology analyzer. Plasma was separated by centrifugation and stored at − 80 °C until analysis. Plasma EPO levels were determined spectrophotometrically using a Multiskan GO microplate spectrophotometer (Thermo Scientific, Vantaa, Finland) and a Rat EPO ELISA kit (Elabscience^®^, Houston, TX, USA), following the manufacturer’s instructions. Inter-experiment variability in absolute plasma EPO values was observed, likely reflecting differences in experimental batches and assay conditions. For example, the plasma EPO concentration in the anemic control group shown in Fig. 8 (173 ± 62 pg/mL) was lower than that observed in Fig. 7 (403 ± 101 pg/mL). To minimize potential confounding effects from such inter-assay or inter-batch variability, all comparisons of plasma EPO levels were therefore performed within the same experimental cohort, and no direct comparisons were made between different experiments.

### Statistical Analysis

Data are presented as mean ± SD. Differences were analyzed by one-way ANOVA with post-hoc tests; correlations used Pearson’s coefficient. *p* < 0.05 was considered significant.

## Results

### Preparation of Oyster Tissue with Different Zinc Levels

**Fig. 1:**
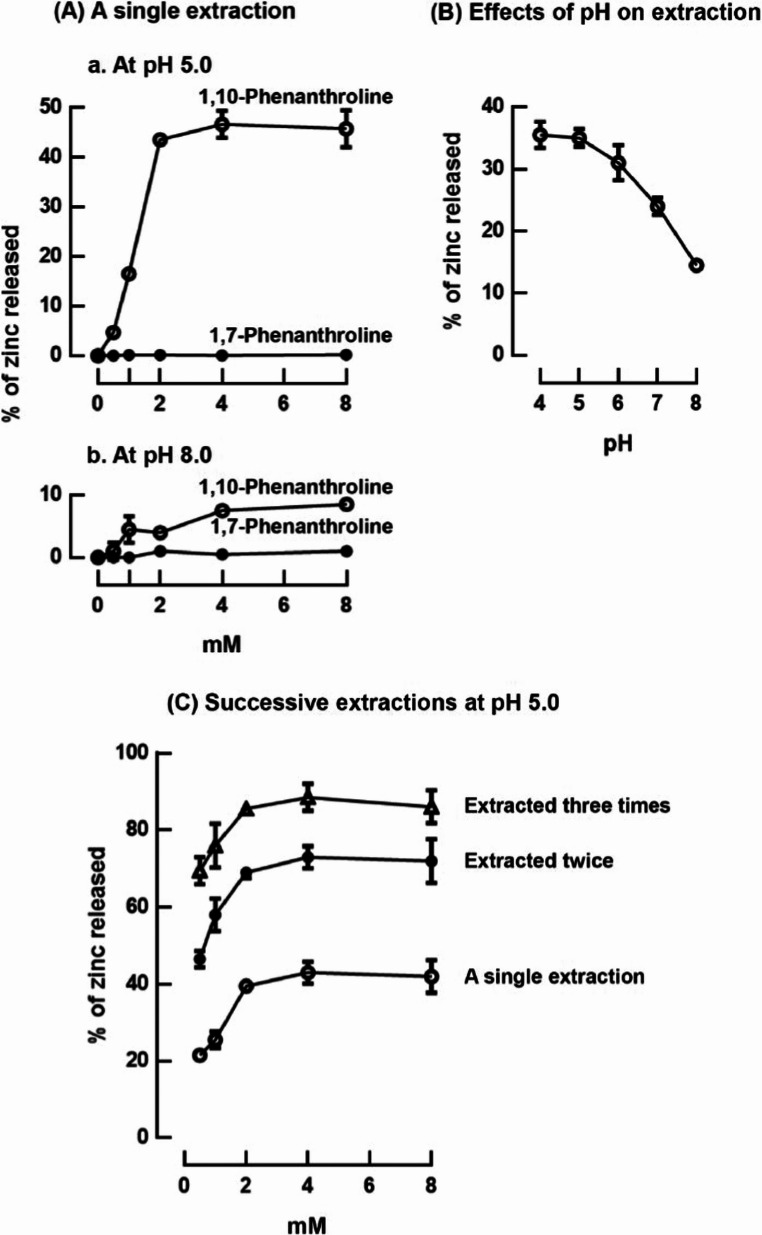
Optimization of zinc removal from oyster tissue using 1,10-phenanthroline. (**A**) Effect of 1,10-phenanthroline concentration (0.5–8 mM) on zinc removal efficiency at pH 5.0 and pH 8.0, compared with the structural analog 1,7-phenanthroline. (**B**) Zinc extraction efficiency of 4 mM 1,10-phenanthroline at different pH values (4–8). (**C**) Cumulative zinc removal from oyster tissue following sequential extractions with 4 mM 1,10-phenanthroline at pH 5.0. Data are presented as mean ± SD (n=3)

To directly evaluate the role of zinc in the anti-anemic activity of oyster tissue, 1,10-phenanthroline—a chelator known to selectively remove catalytic Zn²⁺ from metalloproteins while sparing alkaline-earth metals [30]—was used. This reagent forms soluble Zn–phenanthroline complexes, thereby effectively stripping zinc from metalloproteins [31, 32]. Figure 1 summarizes the optimization of zinc removal from oyster tissue. At equivalent reagent concentrations (0.5–8 mM), zinc extraction was consistently more efficient at pH 5.0 than at pH 8.0 (Fig. 1A). In contrast, the structural analog 1,7-phenanthroline did not extract zinc, confirming the specificity of the chelation reaction for zinc. Acidic conditions (pH 4–5) yielded significantly higher extraction efficiency than neutral or alkaline conditions (pH 7–8) (Fig. 1B). Under optimized conditions (pH 5.0, 4 mM 1,10-phenanthroline, 1 g tissue per two volumes of solution), approximately 40% of zinc was removed in a single extraction. Sequential extractions further increased zinc removal efficiency, with more than 80% of total zinc removed after three extraction cycles (Fig. 1C). The zinc content of untreated fresh oyster tissue was approximately 90 µg Zn/g fresh tissue. By varying the number of extraction cycles, oyster tissue samples containing graded zinc concentrations (30, 43, 50, 70, and 90 µg Zn/g fresh tissue) were generated for subsequent in vivo experiments.

### Effects of Different Zinc Levels in Oyster Tissue on Recovery From PHZ-induced Anemia

**Fig. 2:**
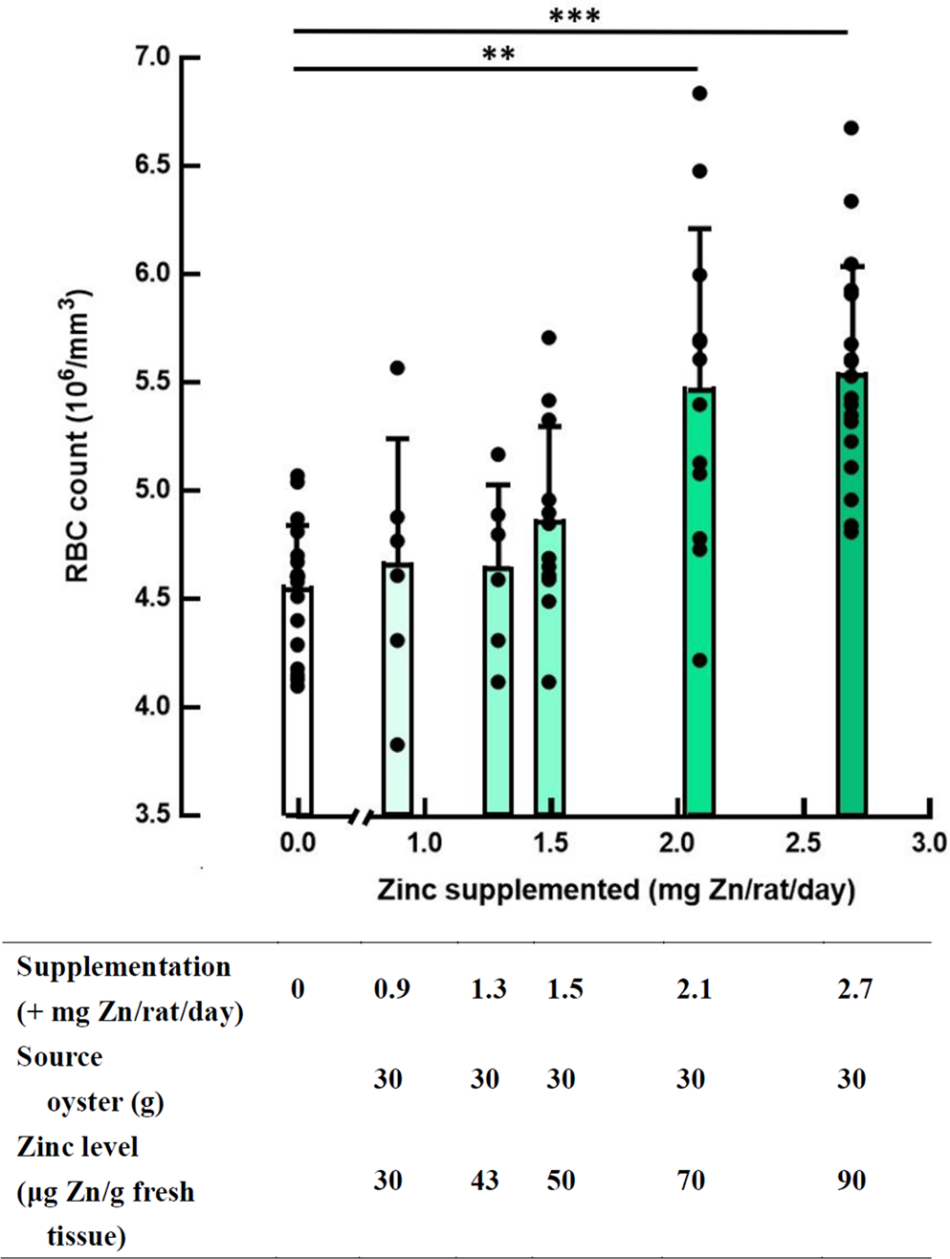
Effect of different zinc levels in oyster tissue on recovery from PHZ-induced anemia. Phenylhydrazine (PHZ)-induced anemic rats were supplemented for four days with oyster tissue equivalent to 30 g fresh oyster/rat/day, containing 0, 0.9, 1.3, 1.5, 2.1, or 2.7 mg Zn/rat/day (n = 12, 6, 6, 12, 12, and 18, respectively). RBC counts were significantly higher in rats receiving 2.1 mg and 2.7 mg Zn/rat/day than in untreated anemic controls. Data are expressed as mean ± SD (**p < 0.01, ***p < 0.001)

PHZ-induced anemic rats were supplemented for four days with equal amounts of oyster tissue (equivalent to 30 g fresh oyster/day in freeze-dried form) received no supplementation (control, 0 mg Zn/rat/day), or containing different zinc levels (0.9, 1.3, 1.5, 2.1, or 2.7 mg Zn/rat/day). As shown in Fig. 2, rats receiving oyster tissue containing 2.1 or 2.7 mg Zn/rat/day exhibited significant recovery of RBC counts compared with untreated anemic controls (*p* < 0.01 and *p* < 0.001, respectively). In contrast, supplementation with lower zinc doses (0.9, 1.3, or 1.5 mg Zn/rat/day) failed to produce a significant hematologic improvement in erythropoietic indices. A dose–response relationship was observed across 0.9–2.1 mg Zn/rat/day. These findings demonstrate that zinc depletion markedly diminishes the anti-anemic activity of oyster tissue and confirm that zinc is the principal active component responsible for correcting PHZ-induced hemolytic anemia. Based on these results, the minimum effective oral zinc dose required to restore erythropoiesis in rats was approximately 2.1 mg Zn/rat/day.

### Comparative Anti-anemic Efficacy of Oyster-derived Zinc and Other Zinc Formulations

**Fig. 3:**
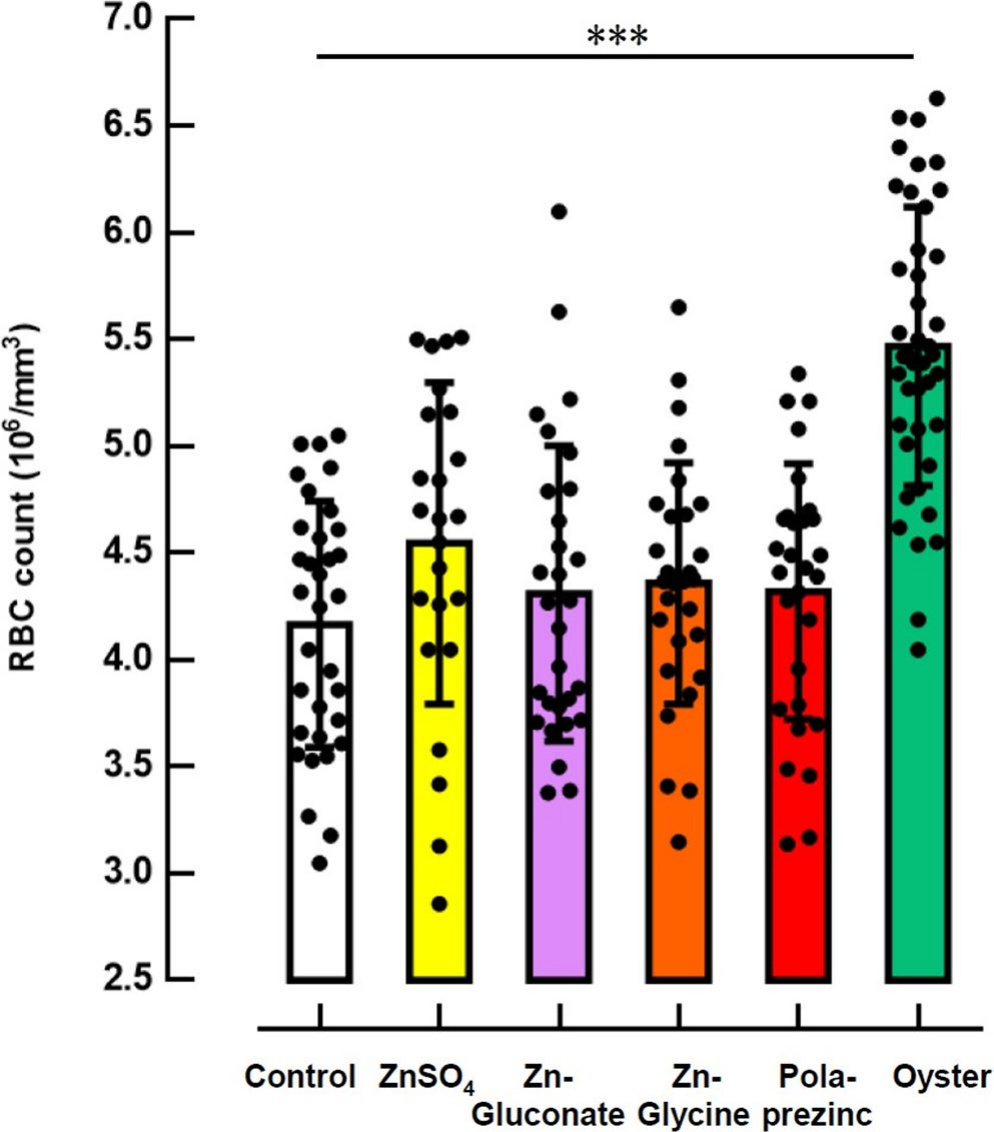
Oyster-derived zinc restores erythropoiesis more effectively than conventional zinc supplements at equal elemental zinc dose. PHZ-induced anemic rats were supplemented for four days with 2.7 mg Zn/rat/day provided as control (no zinc), ZnSO₄, zinc gluconate, zinc glycinate, polaprezinc, or oyster tissue (n = 40, 18, 29, 23, 29, and 40, respectively). Only oyster-derived zinc significantly increased RBC counts compared with untreated the anemic controls. Other zinc formulations did not produce significant improvement. Data are presented as mean ± SD (***p < 0.001)

To compare the anti-anemic efficacy of oyster-derived zinc with commonly used zinc supplements, PHZ-induced anemic rats were supplemented for four days with basal diet only (control), or 2.7 mg Zn/rat/day provided as ZnSO₄, zinc gluconate, zinc glycinate, polaprezinc, or oyster tissue. As shown in Fig. 3, after four days of supplementation, only the group receiving oyster-derived zinc exhibited a significant recovery in RBC counts, reaching 5.47 ± 0.65 × 10⁶/mm³ (78 ± 9% of normal values), compared with 4.17 ± 0.57 × 10⁶/mm³ (59 ± 8% of normal values) in untreated anemic controls (*p* < 0.001). None of the other zinc formulations produced a statistically significant improvement relative to controls. These results demonstrate that oyster-derived zinc provides markedly superior protection against PHZ-induced hemolytic anemia compared with inorganic, organic, or peptide-bound zinc supplements, despite administration of identical zinc doses.

### In Vitro Bioavailability of Oyster-derived Zinc and Other Zinc Sources

#### Simulated Gastrointestinal Zinc Digestibility

**Fig. 4:**
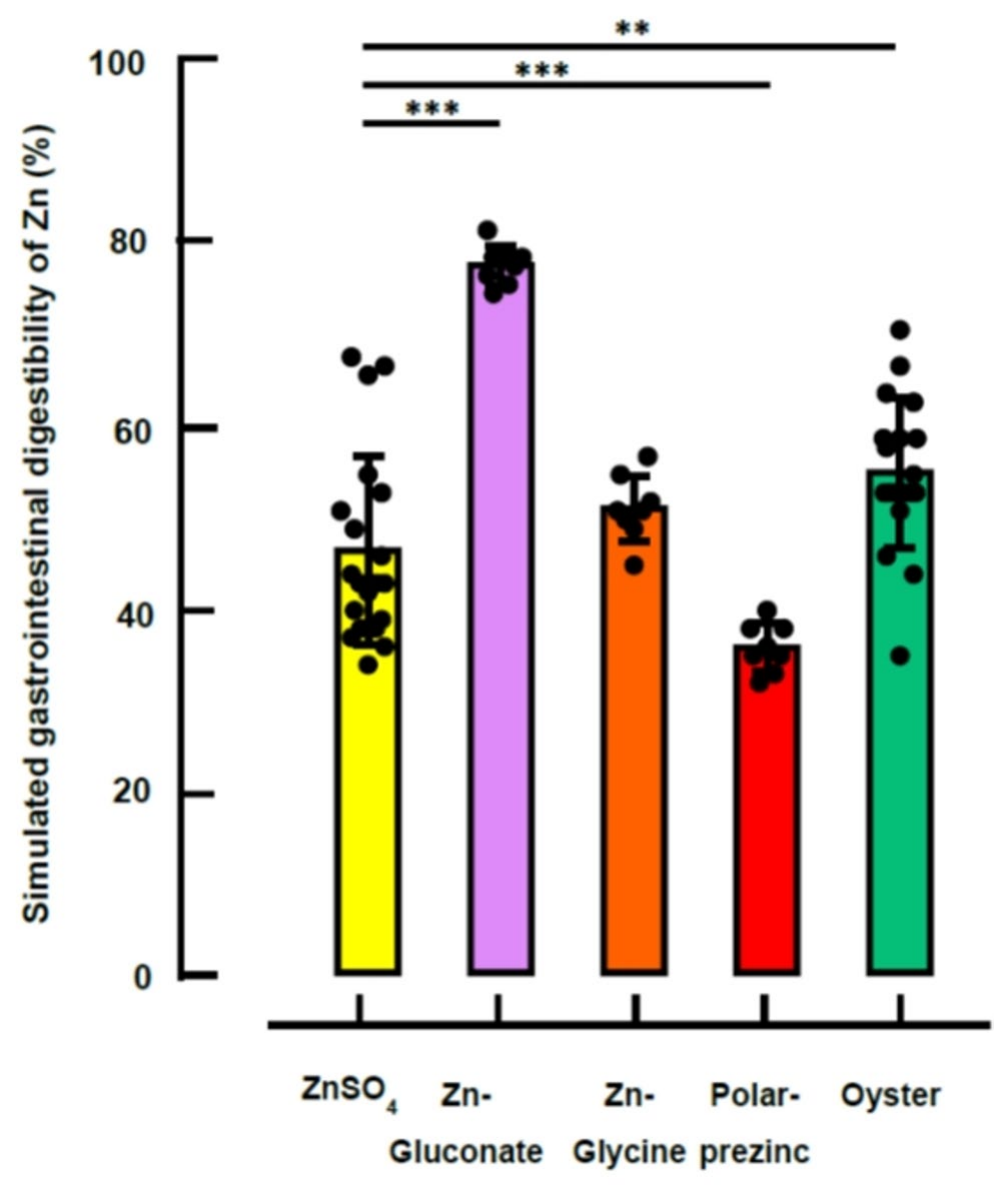
In vitro gastrointestinal digestibility differs among zinc sources. Digestibility (%) was calculated as soluble zinc relative to total zinc after in vitro digestion. Compared with ZnSO₄, zinc gluconate and oyster showed higher digestibility, whereas polaprezinc showed lower digestibility. Data are expressed as mean ± SD (**p < 0.01, ***p < 0.001). Sample sizes were n = 20 (ZnSO₄), 8 (zinc gluconate), 8 (zinc glycinate), 8 (polaprezinc), and 19 (oyster)

Five zinc sources—ZnSO₄, zinc gluconate, zinc glycinate, polaprezinc, and oyster tissue—each containing approximately 2 mg zinc, were subjected to simulated gastrointestinal digestion. Zinc digestibility was calculated as the proportion of zinc present in the digestible fraction relative to total zinc. As shown in Fig. 4, zinc digestibility ranged from 51 ± 4% to 55 ± 8% for zinc glycinate and oyster tissue and was not significantly different ZnSO₄ (47 ± 10%). Zinc gluconate exhibited the highest digestibility (78 ± 2%), whereas polaprezinc showed the lowest digestibility (36 ± 3%).

#### Zinc Absorption in the Caco-2 Cell Model

**Fig. 5:**
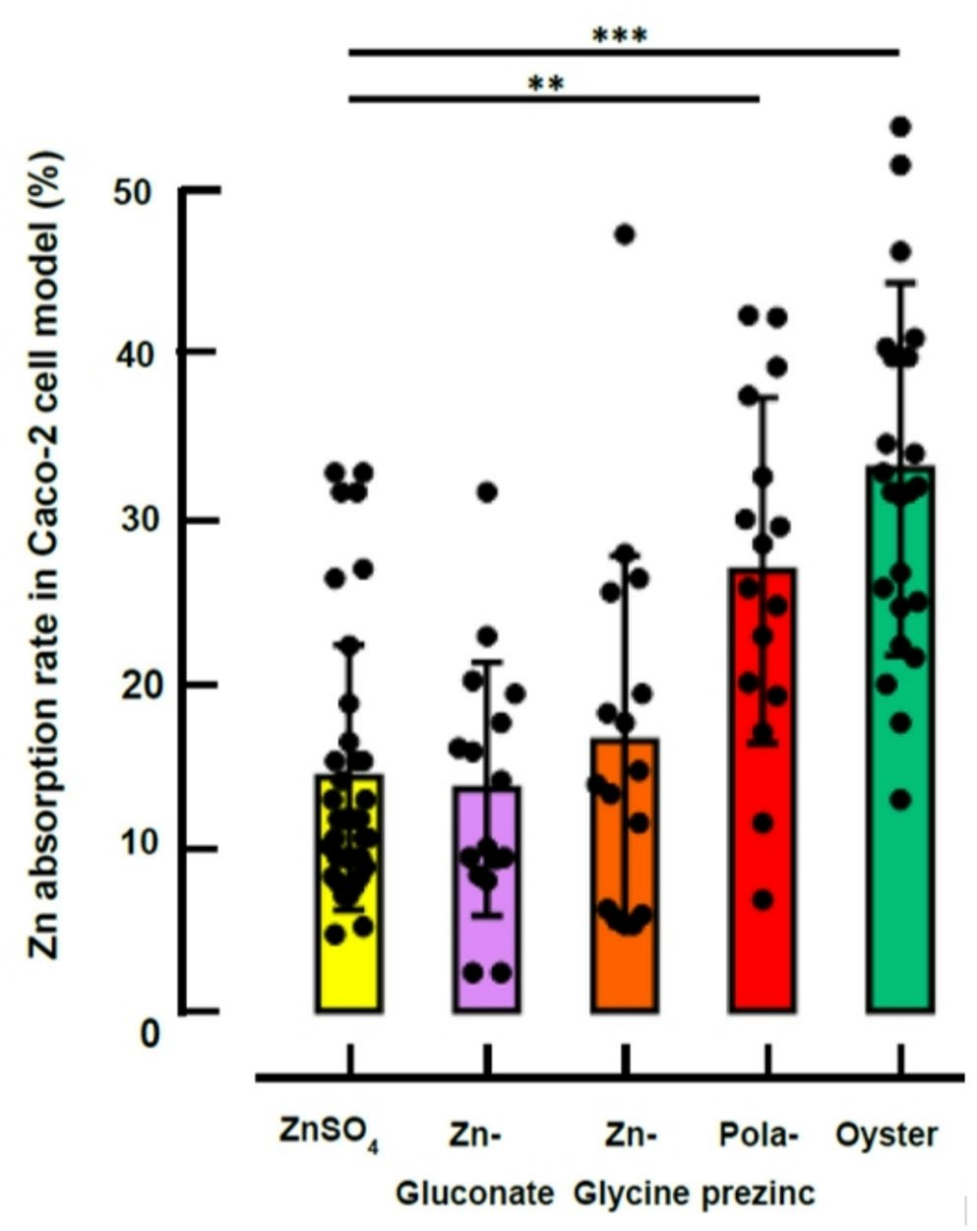
Oyster-derived zinc shows greater transepithelial transport in Caco-2 monolayers than conventional zinc supplements. Digestible fractions (25 µg Zn/mL) were applied apically to differentiated Caco-2 cells cultured in Transwell® inserts. Zinc absorption was quantified as the percentage of basolateral zinc transport. Oyster-derived zinc and polaprezinc showed significantly higher absorption than ZnSO₄ Data are expressed as mean ± SD (**p < 0.01, ***p < 0.001). Sample sizes were n = 37 (ZnSO₄), 16 (zinc gluconate), 16 (zinc glycinate), 16 (polaprezinc), and 24 (oyster)

Digestible fractions were adjusted to 25 µg Zn/mL and applied to differentiated Caco-2 monolayers. As shown in Fig. 5, zinc absorption rates were 15.2 ± 6.9% (ZnSO₄), 14.5 ± 6.6% (zinc gluconate), 17.1 ± 9.6% (zinc glycinate), 25.9 ± 9.0% (polaprezinc), and 31.2 ± 9.7% (oyster), respectively. Oyster-derived zinc exhibited the highest absorption rate, approximately 2.1-fold greater than ZnSO₄ (*p* < 0.001).

#### Overall in Vitro Zinc Bioavailability

Overall in vitro zinc bioavailability—calculated by integrating digestion efficiency and Caco-2 absorption rate—is summarized in Table 1. Oyster-derived zinc displayed the highest overall bioavailability, nearly twofold greater than all other zinc sources tested. Although in vitro digestibility of oyster-derived zinc was comparable to that of ZnSO₄ (Fig. 4), Caco-2 transport assays demonstrated markedly higher intestinal zinc absorption from oyster tissue (Fig. 5), indicating that differences in overall bioavailability are primarily driven by post-digestive uptake rather than luminal solubility. This enhanced bioavailability likely explains the superior anti-anemic efficacy observed in vivo: at 2.7 mg Zn/rat/day, oyster supplementation corrected PHZ-induced anemia, whereas an equivalent zinc dose from other sources did not (Fig. 3). Based on absorption estimates, only approximately 1.4 mg Zn/rat/day was absorbed from non-oyster zinc sources, which falls below the ~ 2.1 mg Zn threshold required for anemia correction (Fig. 2).

**Table 1:** In vitro bioavailability of oyster-derived zinc and other zinc sources. (In Vitro Bioavailability = Simulated gastrointestinal digestibility of Zn× Zn absorption rate in Caco-2 cell model)

Zn sources	Digestibility (%)	Caco-2 Absorption (%)	In vitro Bioavailability (%)	Ratio
ZnSO₄	47	15.2	7.14	0.42
Zn-gluconate	78	14.5	11.3	0.66
Zn-glycine	51	17.1	8.72	0.51
Polaprezinc (Zn-L carnosine)	36	25.9	9.32	0.54
Oyster	55	31.2	17.2	1.00

### Relationship Between RBC Counts and Plasma EPO Levels in Zinc-supplemented Rats

#### Supplementation With Different Zinc Levels in the Same Amount of Oyster Tissue

**Fig. 6:**
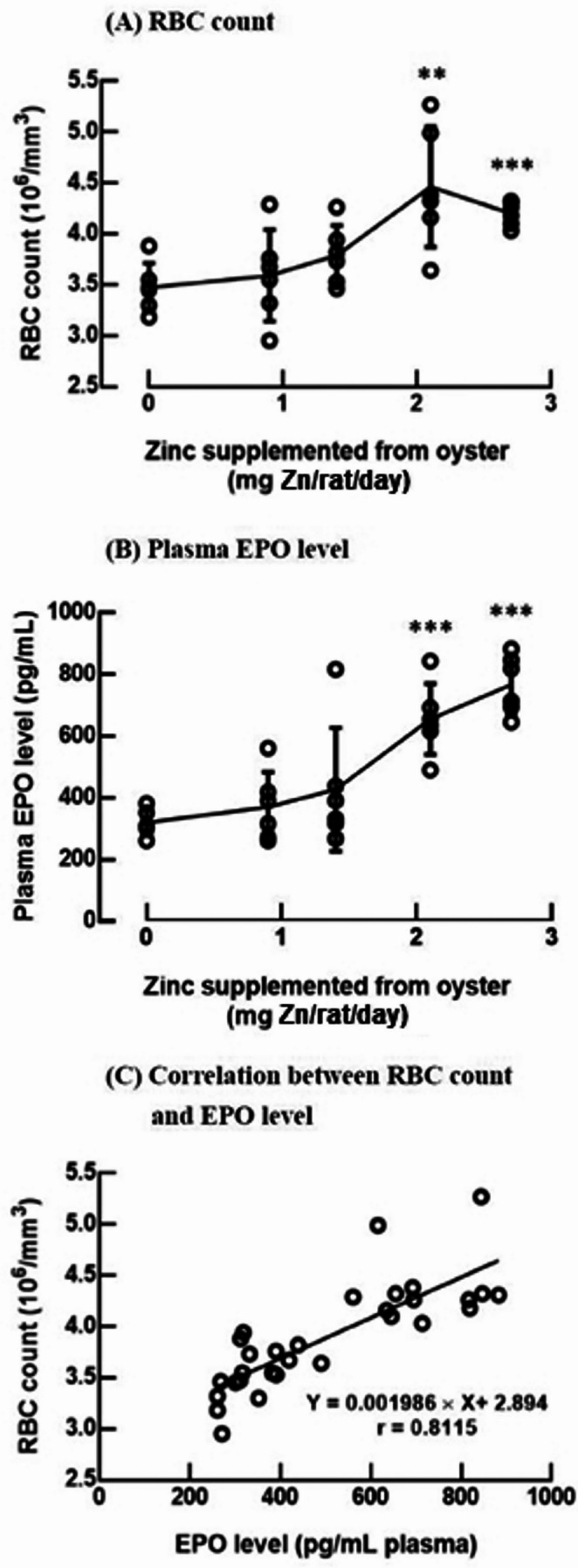
Effect of different zinc levels in oyster tissue on RBC counts and plasma erythropoietin (EPO) levels in PHZ-induced anemic rats. PHZ-induced anemic rats were supplemented for four days with the same amount of oyster tissue (equivalent to 30 g fresh oyster/rat/day) containing 0, 0.9, 1.4, 2.1, or 2.7 mg Zn/rat/day (n = 6 per group). (**A**) RBC counts across zinc-supplementation groups. (**B**) Plasma EPO levels across zinc-supplementation groups. (**C**) Correlation between RBC counts and plasma EPO levels. Data are presented as mean ± SD. Statistical significance was determined relative to the control group (**p < 0.01, ***p < 0.001)

As shown in Fig. 6A, PHZ-induced anemic rats supplemented with oyster tissue containing 2.1 or 2.7 mg Zn/rat/day exhibited significantly higher RBC counts compared with controls. A similar pattern was observed for plasma EPO levels (Fig. 6B), with both zinc doses producing significant elevations relative to controls. Importantly, RBC counts were strongly correlated with plasma EPO concentrations (Fig. 6C), indicating that zinc-induced EPO production closely parallels the restoration of erythropoiesis.

#### Supplementation With Oyster-derived Zinc or Other Zinc Sources

**Fig. 7:**
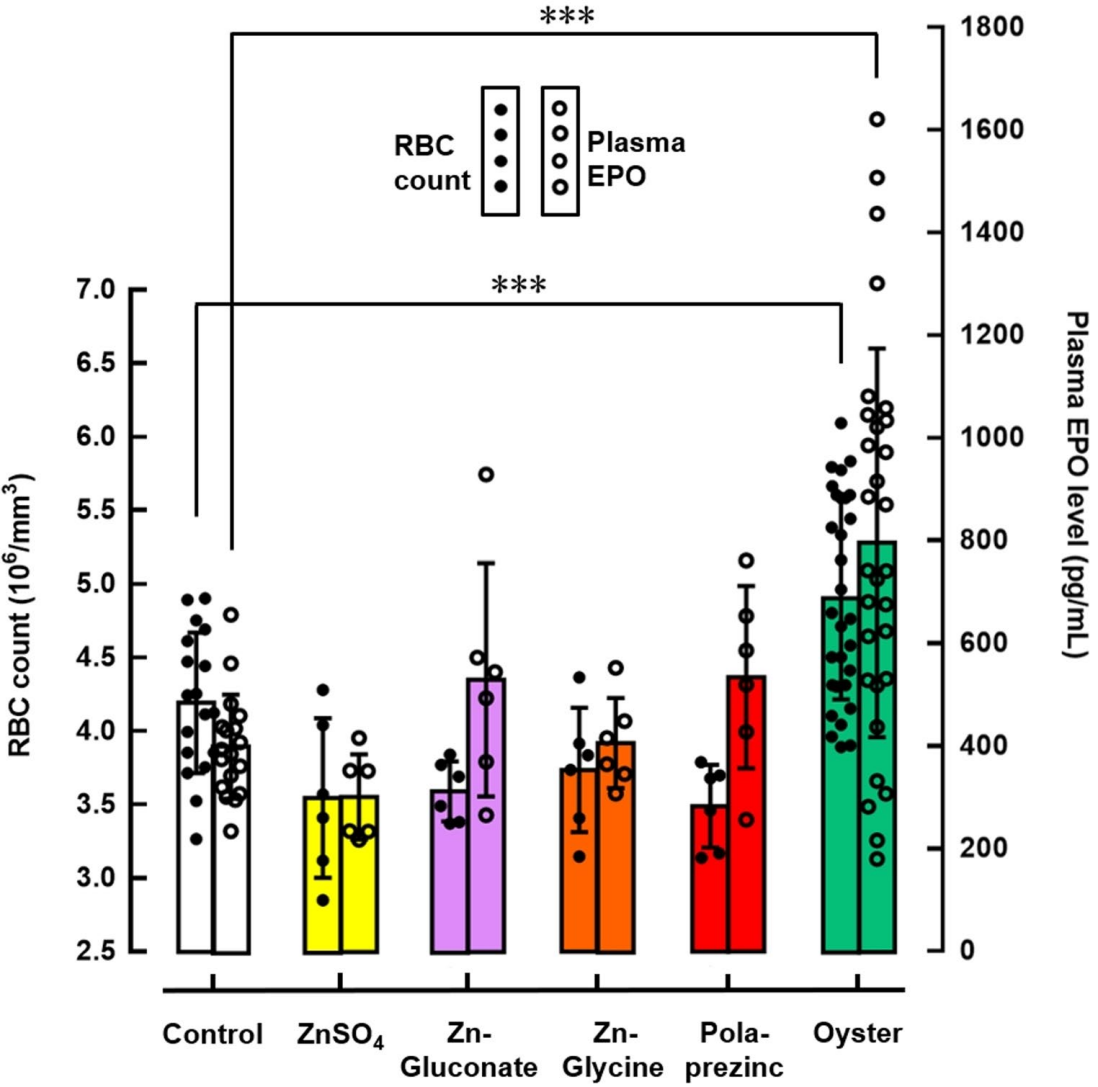
Only oyster-derived zinc increases plasma EPO and RBC recovery at equal elemental zinc dose. PHZ-induced anemic rats were supplemented for four days with 2.7 mg Zn/rat/day provided as control (no zinc), ZnSO₄, zinc gluconate, zinc glycinate, polaprezinc, or oyster tissue (n = 18, 6, 6, 6, 6, and 30, respectively). Only oyster-derived zinc significantly increased both RBC counts and plasma EPO levels compared with untreated anemic controls. Data are expressed as mean ± SD (***p < 0.001)

To further assess the specificity of EPO induction, RBC counts and plasma EPO levels were measured in rats supplemented with oyster-derived zinc or other zinc formulations. As shown in Fig. 7, only rats receiving oyster-derived zinc demonstrated significant recovery of RBC counts relative to untreated anemic controls (*p* < 0.001). Correspondingly, plasma EPO levels increased from 403 ± 101 pg/mL in anemic controls to 798 ± 438 pg/mL in the oyster-derived zinc group (*p* < 0.001), representing an approximate twofold increase. None of the other zinc formulations elicited a comparable EPO response. These findings indicate that zinc delivered within the natural oyster matrix preferentially stimulates endogenous EPO production, likely reflecting superior zinc bioavailability.

### Minimum Effective Oral Zinc Dose From ZnSO₄ Required to Restore Erythropoiesis in Rats

**Fig. 8:**
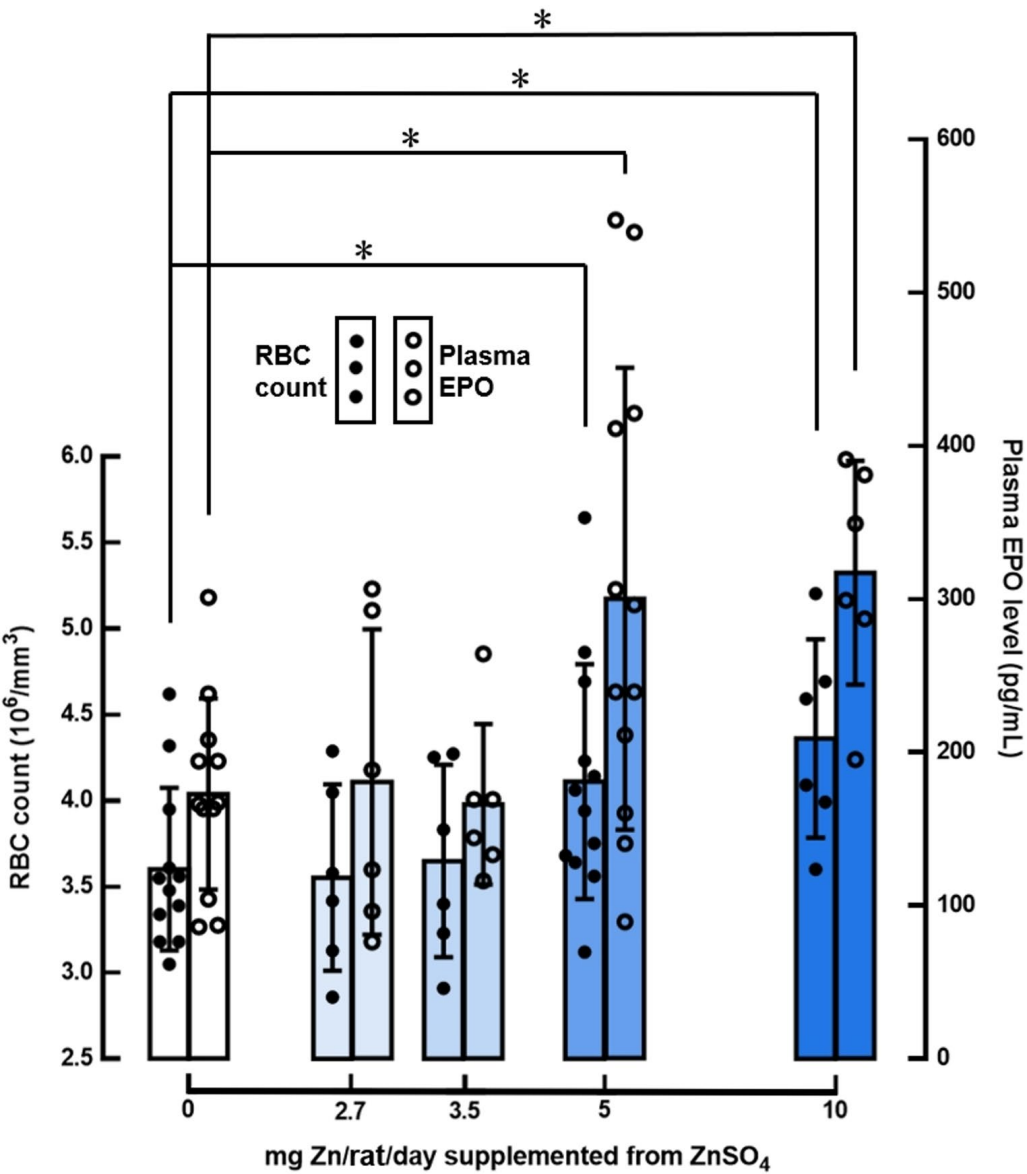
Higher oral ZnSO₄ dose is required to increase RBC counts and plasma EPO in PHZ-induced anemia. Because oral ZnSO₄ exhibits lower bioavailability than oyster-derived zinc (Table 1), higher ZnSO₄ doses were tested to determine the minimum oral dose required to reach an erythropoietic effect. PHZ-induced anemic rats were supplemented for four days with ZnSO₄ providing 0, 2.7, 3.5, 5.0, or 10 mg Zn/rat/day (n = 12, 6, 6, 12, and 6, respectively). Both RBC counts and plasma EPO levels were significantly higher in rats receiving 5.0 mg and 10 mg Zn/rat/day compared with untreated anemic controls. Data are expressed as mean ± SD (*p < 0.05)

As shown in Fig. 8, the minimum effective oral zinc dose from ZnSO₄ required to restore erythropoiesis in PHZ-induced anemic rats was 5.0 mg Zn/rat/day, which is approximately 2.4-fold greater than the 2.1 mg Zn/rat/day required for oyster-derived zinc (Fig. 2). This observation is consistent with the finding that oyster-derived zinc exhibited approximately 2.4-fold higher bioavailability than ZnSO₄ (Table 1). Consistently, plasma EPO levels increased from 173 ± 62 pg/mL in anemic controls to 300 ± 151 pg/mL in rats supplemented with 5.0 mg Zn/rat/day from ZnSO₄ (*p* < 0.05), representing an approximate 1.8-fold increase.

## Discussion

### Oyster-derived Zinc as a Superior Zinc Source for Nutritional Supplementation

The present study demonstrates that oyster-derived zinc exhibits higher bioavailability and greater anti-anemic efficacy than several commonly used zinc supplements under the experimental conditions tested. Although whole oyster tissue was used as the dietary intervention, multiple independent lines of evidence indicate that zinc is the principal determinant of the observed erythropoietic effect. Selective depletion of zinc from oyster tissue using 1,10-phenanthroline abolished its anti-anemic activity (Fig. 2), strongly supporting zinc as essential for the erythropoietic activity associated with oyster supplementation. This conclusion is further supported by our previous findings showing that hard clams (*Meretrix lusoria*), which are nutritionally comparable to oysters but contain substantially lower zinc levels, failed to improve RBC counts in anemic rats and therefore served as an effective placebo control [6].

When equivalent amounts of elemental zinc were administered, oyster-derived zinc consistently produced greater recovery from anemia than inorganic, organic, or peptide-bound zinc formulations (Fig. 3). This superior biological efficacy is closely linked to zinc bioavailability. In bioavailability assays, zinc derived from oyster tissue exhibited approximately two-fold higher intestinal absorption than all other zinc formulations tested (Table 1). Although recent clinical reviews have suggested that certain organic zinc salts, including zinc glycinate and zinc gluconate, may display improved absorption compared with inorganic zinc salts [33], our data demonstrate that zinc delivered within the natural oyster matrix achieves significantly greater intestinal uptake than these commonly used supplements. Consistent with this observation, the minimum zinc dose required to restore erythropoiesis was markedly lower for oyster-derived zinc (2.1 mg Zn/rat/day) than for ZnSO₄ (5.0 mg Zn/rat/day), indicating a substantially higher biological efficiency of zinc when delivered from oysters.

Beyond dose comparisons, enhanced intestinal uptake appears central to the observed biological differences. Caco-2 transport assays demonstrated markedly greater transepithelial zinc transport from oyster tissue than from inorganic, organic, or peptide-bound zinc formulations. This improved intestinal absorption was mirrored in vivo, where only oyster-derived zinc significantly increased plasma EPO levels and restored red blood cell counts in PHZ-induced hemolytic anemia (Figs. 6 and 7). These findings support the concept that zinc bioavailability, rather than administered dose alone, is a critical determinant of erythropoietic efficacy.

From a nutritional perspective, these results highlight oyster-derived zinc as an effective strategy for alleviating zinc deficiency and zinc-responsive anemia. Given the high zinc content of oysters and their superior bioavailability, relatively small amounts of oyster-derived zinc may be sufficient to achieve physiological benefit. Moreover, oysters are widely consumed and, in many regions, represent an economically accessible dietary source of zinc. Collectively, our findings support the concept that delivering zinc within a natural food matrix can enhance its biological effectiveness and may offer advantages over conventional zinc supplements in nutritional interventions.

### Zinc as a Regulator of Erythropoiesis

Iron deficiency is widely recognized as the predominant nutritional cause of anemia, however, the role of zinc in erythropoiesis has received comparatively less attention in nutritional research. Accumulating evidence indicates that zinc functions not only as a structural and catalytic cofactor, but also as a regulatory element influencing hematopoietic signaling pathways. It is known that zinc supplementation stimulates erythropoiesis, increases reticulocyte counts, and restores hemoglobin concentrations across multiple experimental anemia models [3–6]. Based on these observations, we previously proposed a two-stage mechanism of zinc-induced erythropoiesis, in which zinc first stimulates endogenous EPO production, followed by EPO-mediated activation of erythroid progenitors [5]. The present study provides additional support for this model. Across zinc-supplemented groups, RBC counts were strongly correlated with plasma EPO concentrations (Fig. 6C), indicating that EPO induction closely parallels erythropoietic recovery. Notably, only rats receiving oyster-derived zinc exhibited significant increases in both RBC counts and plasma EPO levels compared with untreated anemic controls (Fig. 7). None of the other zinc formulations elicited a comparable EPO response, despite administration of equivalent elemental zinc doses. These findings suggest that, under anemic conditions, a threshold level of bioavailable zinc is required to activate endogenous EPO production. Analysis of plasma EPO concentrations indicates that erythropoietic recovery was accompanied by an approximately two-fold increase in EPO levels relative to the anemic state (Figs. 7 and 8). Although this observation does not establish a fixed physiological requirement, it provides a useful biochemical reference linking zinc bioavailability to erythropoietic signaling. Collectively, these results reinforce the conclusion that the erythropoietic activity of oyster-derived zinc is mediated, at least in part, through zinc-dependent stimulation of EPO production.

### Translational Dose Considerations and Clinical Feasibility

Defining a safe and effective zinc dose is essential for clinical translation. In the present study, the minimum oral zinc dose required to correct anemia in rats was 2.1 mg Zn/rat/day, corresponding to approximately 6.4 mg Zn/kg/day based on an average body weight of 0.33 kg. This dose is far below established toxicological thresholds, including the reported oral NOAEL (No Observed Adverse Effect Level) for zinc in rats (≈ 234–243 mg Zn/kg/day) [34] providing a nearly 40-fold safety margin. Using standard body-surface-area conversion with FDA-recommended Km factors [35], this rat dose translates to a human-equivalent dose (HED) of approximately 1.03 mg Zn/kg/day, corresponding to 62 mg/day for a 60-kg adult or 72 mg/day for a 70-kg adult. Notably, these values fall within the range of zinc doses already employed in clinical studies of chronic kidney disease and dialysis patients, where 34–100 mg/day of elemental zinc—administered as zinc sulfate, zinc acetate, zinc gluconate, zinc glycinate, or polaprezinc—has been reported to improve hematologic or related outcomes [36–38]. Even higher doses (100 mg/day) have been reported to be well tolerated in short-term studies [39, 40].

Thus, the HED derived from our animal data aligns closely with clinically tested zinc doses. Given the superior bioavailability of oyster-derived zinc demonstrated in the present study, the effective clinical dose may be lower than that predicted by body-surface-area scaling alone. Collectively, the favorable safety margin, concordance with existing clinical dosing experience, and enhanced bioavailability support the feasibility of evaluating oyster-derived zinc in clinical trials aimed at correcting anemia, particularly in settings where EPO responsiveness is impaired or oral supplementation is preferred.

### Possible Mechanisms Underlying the Superior Bioavailability of Oyster-derived Zinc

Zinc in oysters exists predominantly in protein- or peptide-associated forms. A 43-kDa zinc-binding protein has been identified in oyster tissue [41], and several zinc-rich peptides have been isolated [42–44]. These forms may facilitate intestinal uptake through interactions with zinc-specific transporters such as ZIP4 and peptide transport systems such as PEPT1. In addition, peptide-bound zinc may be less susceptible to inhibition by dietary antagonists such as phytates [45–46]. Although transporter expression was not directly examined in this study, these proposed mechanisms are consistent with the enhanced transepithelial transport observed in vitro and the improved physiological outcomes observed in vivo.

### To Alleviate Anemia, Zinc Supplementation or Iron supplementation?

Anemia arises from heterogeneous etiologies, including nutritional deficiencies (e.g., iron deficiency) and disease-associated conditions such as chronic kidney disease (CKD). Iron supplementation remains the standard therapy for iron-deficiency anemia. In contrast, anemia associated with CKD is largely driven by impaired EPO production, and iron supplementation alone may be insufficient without addressing reduced EPO activity.

Zinc supplementation alone would not be expected to correct classical iron-deficiency anemia in the absence of iron repletion, just as iron supplementation alone may not fully resolve anemia primarily driven by impaired EPO production. These distinctions emphasize that therapeutic strategies should be aligned with the underlying pathophysiology of anemia.

### Limitations and Future Prospects

A limitation of the present study is that the specific zinc-binding protein(s) or peptide(s) responsible for the enhanced bioavailability remain to be identified. It is unclear whether the bioactive species correspond to previously reported zinc-binding proteins or peptides isolated from oysters. In addition, mechanistic insights into specific zinc transport pathways were not directly investigated.

Future studies examining zinc transporter expression, peptide–zinc structural characterization, and transporter inhibition assays would provide further mechanistic clarification. Identifying the molecular complexes responsible for enhanced zinc absorption and erythropoietic activity would deepen understanding of zinc biology and may facilitate the development of optimized zinc-based nutritional or therapeutic formulations.

## Supplementary Information

Below is the link to the electronic supplementary material.

Supplementary Material 1 (DOCX 14.0 KB)

## Data Availability

The data supporting the findings of this study are available from the corresponding authors upon reasonable request.
