# Supplementary material for: Oyster-derived Zinc Exhibits Superior Anti-anemic Efficacy and Bioavailability Compared with Conventional Zinc Supplements
Source: Biol Trace Elem Res. 2026 Mar 25;204(8):5830–42. doi: 10.1007/s12011-026-05073-x (PMC13369750; doi:10.1007/s12011-026-05073-x)
Supplement: Supplementary file 1 — Supplementary Material 1 (DOCX 14.0 KB) [file 12011_2026_5073_MOESM1_ESM.docx]

Supplemental Table 1. Nutrient Composition of the basal diet (milk fat globule (MFG) diet)

| **Nutrition** | **Composition** | **Energy%** |
| --- | --- | --- |
| Vitamin A | 29,000 IU/kg |  |
| Vitamin D3 | 6.5 g/kg |  |
| Vitamin E | 212 mg/kg |  |
| Vitamin B1 | 44 mg/kg |  |
| Vitamin B2 | 30.1 mg/kg |  |
| Vitamin B6 | 10.5 mg/kg |  |
| Moisture | 79.0 g/kg |  |
| Crude Protein | 229.0 g/kg | 25.7 |
| Crude Fat | 54.0 g/kg | 13.6 |
| Crude Ash | 62.0 g/kg |  |
| Crude Fiber | 34.0 g/kg |  |
| Nitrogen Free Extract | 542.0 g/kg | 60.7 |
| Calorie | 3570 Kcal/kg |  |
| Total | 1000 g |  |

Dietary composition provided by manufacturer (Oriental Yeast Co., Ltd., Tokyo, Japan). The micronutrient profile, including vitamin and mineral mixtures, was strictly formulated according to AIN-93G specifications.
